# A Rolling Stone Gathers No Moss

**Published:** 1875-12

**Authors:** 


### Editorial.

A ROLLING STONE GATHERS NO MOSS.—We have many inquiries made of us for an "opening" for the profitable practice of medicine. With not a few practitioners, there is a feeling of perpetual unrest; they seem to be annually moving their locations, or contemplating removal. All this, we think, is ill-advised, and inures neither to the advantage of the profession nor to the benefit of the community.

Unquestionably a physician may outgrow the community in which he labors and may properly and advantageously seek a larger field. Such cases, however, of *real growth* are exceptional, and bear no comparison to the many who exhibit a restless dissatisfaction with the things that *are* and look longingly after that which *seems* to be in the far off distance. This feeling is perhaps natural, and more or less pervades all classes and callings; but in few occupations is its indulgence more destructive of true prosperity than in our own.

There is no business which is so dependent for its advancement upon public *confidence* as the practice of medicine. Confidence is notoriously a plant of slow growth, and does not bear repeated transplanting. If it be diligently cultivated and watered by daily and nightly toil, slowly but surely it takes deep root, silently and unobserved for a time, away in the bosom of mother earth. By careful, untiring nurture, in due season the branches begin to expand, little by little at first, but with a constantly increasing rapidity, until the careworn laborer, passing the zenith and beginning to descend upon the farther side of the hill, is able to shelter himself comfortably and happily beneath its widely extended shade. Let the tender twig be transplanted from its native soil, and ere the leaf has fallen before a second frost, again and again uprooted, and no gift of prophecy is needed to foresee a dwarfed and stunted growth, or mayhap untimely death and decay.

No honest and industrious physician, be he ever so humble in his capacity, can follow his vocation diligently for a twelvemonth in any locality and fail to impress *somebody* with a just estimate of his services. If he will but content himself with the progress made, and continue his efforts patiently and perseveringly, he will surely add friend to friend and patron to patron, until at last peace and plenty shall smile upon his honest and earnest endeavors.

It is no uncommon error for medical men to find the widest discrepancy existing between the esteem in which they are held by the community and the self-estimate of their own capacity. Such men are the victims of a cold, unfeeling world. They are unappreciated by the public. They become per consequence soured with the world, and very naturally resent the injustice, the blindness and ignorance of mankind, who fail to discern their supposed merit and capacity. We verily believe that these professional, self-constituted martyrs to the coldness, blindness, and ignorance, which surrounds and engulphs them, are but too often the architects of their own mal-fortune. Nobody chooses a soured misanthrope for his companion; nobody takes a mountain of self-conceit for his bosom friend.

Whilst we cannot subscribe fully to the sentiment "*vox populi, vox dei*," we are nevertheless of those who believe that the deliberate verdict of the community upon the capacity and merit of those who serve it is in the main just and equitable. Quite sure are we that it is the part of wisdom in a medical man to bow cheerfully to such verdict, and, rather than waste himself in vain endeavors to impeach the court which judges him, he should strive to secure a renewed and more favorable hearing by reviewing his own ground and rendering himself more worthy of it.

There are some among us who have mistaken their calling. Some of these—wise men—cheerfully accept the verdict of the community, and retire to the plough, to the anvil, to the counter, to the bar, or to the desk, and finding the congenial atmosphere, lead useful and successful lives, and are respected and honored by their fellows. Others obstinately wrapping themselves in their mantle of self conceit, and in sour and dejected self martyrdom, roam the earth, making their subsistence scantily upon the crumbs, whilst they appeal to one community after another for the redress of their fancied wrongs, but, alas! meeting everywhere the same chilling response, "*tekel, tekel.*"

Is it an "opening" that is wanted? If we discern aright it is *men*, true men, honest, industrious, laboring men, men of energy, men of vim, men of "pluck" that constitute the real want. In this broad land we see openings everywhere for such men; but we know of no opening anywhere for some men we wot of. We believe it is Daniel Webster who is to be credited with the remark that the professions everywhere are crowded to death down stairs, but there is always plenty of room in the upper stories. Whether Webster or not matters little, no man, be he ever so great, need feel ashamed of having made so valuable a discovery. It is pluck to climb that our correspondents need, and not openings to fall into.

